# Temporal trends in diabetic ketoacidosis at diagnosis of paediatric type 1 diabetes between 2006 and 2016: results from 13 countries in three continents

**DOI:** 10.1007/s00125-020-05152-1

**Published:** 2020-05-08

**Authors:** Valentino Cherubini, Julia M. Grimsmann, Karin Åkesson, Niels H. Birkebæk, Ondrej Cinek, Klemen Dovč, Rosaria Gesuita, John W. Gregory, Ragnar Hanas, Sabine E. Hofer, Reinhard W. Holl, Craig Jefferies, Geir Joner, Bruce R. King, Elizabeth J. Mayer-Davis, Alexia S. Peña, Birgit Rami-Merhar, Ulrike Schierloh, Torild Skrivarhaug, Zdenek Sumnik, Jannet Svensson, Justin T. Warner, Nataša Bratina, Dana Dabelea

**Affiliations:** 1 Division of Pediatric Diabetology, Department of Women’s and Children’s Health, Salesi Hospital, Ancona, Italy; 2 Institute of Epidemiology and Medical Biometry, ZIBMT, University of Ulm, Albert-Einstein-Allee 41, 89081 Ulm, Germany; 3 German Center for Diabetes Research (DZD), Munich-Neuherberg, Germany; 4 Department of Biomedical and Clinical Sciences, Linköping University, Linköping, Sweden; 5 Department of Pediatrics, Ryhov County Hospital, Jönköping, Sweden; 6 Department of Pediatrics, Aarhus University Hospital, Aarhus, Denmark; 7 Department of Pediatrics, 2nd Faculty of Medicine, Charles University in Prague and Motol University Hospital, Prague, Czechia; 8 Department of Paediatric Endocrinology, Diabetes and Metabolic Diseases, UMC – University Children’s Hospital and Faculty of Medicine, Ljubljana, Slovenia; 9 Centre of Epidemiology and Biostatistics, Polytechnic University of Marche, Via Tronto 10/a, 60020 Ancona, Italy; 10 Division of Population Medicine, School of Medicine, Cardiff University, Cardiff, UK; 11 Department of Pediatrics, NU Hospital Group, Uddevalla, Sweden; 12 Sahlgrenska Academy, Institute of Clinical Sciences, Gothenburg University, Gothenburg, Sweden; 13 Department of Pediatrics 1, Medical University of Innsbruck, Innsbruck, Austria; 14 Department of Endocrinology, Starship Children’s Health, Auckland, New Zealand; 15 Division of Paediatric and Adolescent Medicine, Oslo University Hospital, Oslo, Norway; 16 Institute of Clinical Medicine, University of Oslo, Oslo, Norway; 17 Department of Paediatric Diabetes, John Hunter Children’s Hospital, Faculty of Medicine, University of Newcastle, Newcastle, NSW Australia; 18 Departments of Nutrition and Medicine, University of North Carolina, Chapel Hill, NC USA; 19 Paediatrics, Robinson Research Institute, The University of Adelaide, Adelaide, SA Australia; 20 Department of Pediatric and Adolescent Medicine, Medical University of Vienna, Vienna, Austria; 21 DECCP, Clinique Pédiatrique, Centre Hospitalier, Luxembourg, Luxembourg; 22 Department of Pediatrics and Adolescent Medicine, Copenhagen University Hospital, Herlev, Denmark; 23 Department of Child Health, University Hospital of Wales, Cardiff, UK; 24 Lifecourse Epidemiology of Adiposity and Diabetes (LEAD) Center, Colorado School of Public Health, University of Colorado Anschutz Medical Campus, Aurora, CO USA

**Keywords:** Children with diabetes, Complications, Diabetic ketoacidosis, Diagnosis of diabetes, Epidemiology, Type 1 diabetes

## Abstract

**Aims/hypothesis:**

The aim of this work was to evaluate geographical variability and trends in the prevalence of diabetic ketoacidosis (DKA), between 2006 and 2016, at the diagnosis of childhood-onset type 1 diabetes in 13 countries over three continents.

**Methods:**

An international retrospective study on DKA at diagnosis of diabetes was conducted. Data on age, sex, date of diabetes diagnosis, ethnic minority status and presence of DKA at diabetes onset were obtained from Australia, Austria, Czechia, Denmark, Germany, Italy, Luxembourg, New Zealand, Norway, Slovenia, Sweden, USA and the UK (Wales). Mean prevalence was estimated for the entire period, both overall and by country, adjusted for sex and age group. Temporal trends in annual prevalence of DKA were estimated using logistic regression analysis for each country, before and after adjustment for sex, age group and ethnic minority status.

**Results:**

During the study period, new-onset type 1 diabetes was diagnosed in 59,000 children (median age [interquartile range], 9.0 years [5.5–11.7]; male sex, 52.9%). The overall adjusted DKA prevalence was 29.9%, with the lowest prevalence in Sweden and Denmark and the highest in Luxembourg and Italy. The adjusted DKA prevalence significantly increased over time in Australia, Germany and the USA while it decreased in Italy. Preschool children, adolescents and children from ethnic minority groups were at highest risk of DKA at diabetes diagnosis in most countries. A significantly higher risk was also found for females in Denmark, Germany and Slovenia.

**Conclusions/interpretation:**

DKA prevalence at type 1 diabetes diagnosis varied considerably across countries, albeit it was generally high and showed a slight increase between 2006 and 2016. Increased awareness of symptoms to prevent delay in diagnosis is warranted, especially in preschool children, adolescents and children from ethnic minority groups.

**Electronic supplementary material:**

The online version of this article (10.1007/s00125-020-05152-1) contains peer-reviewed but unedited supplementary material, which is available to authorised users.

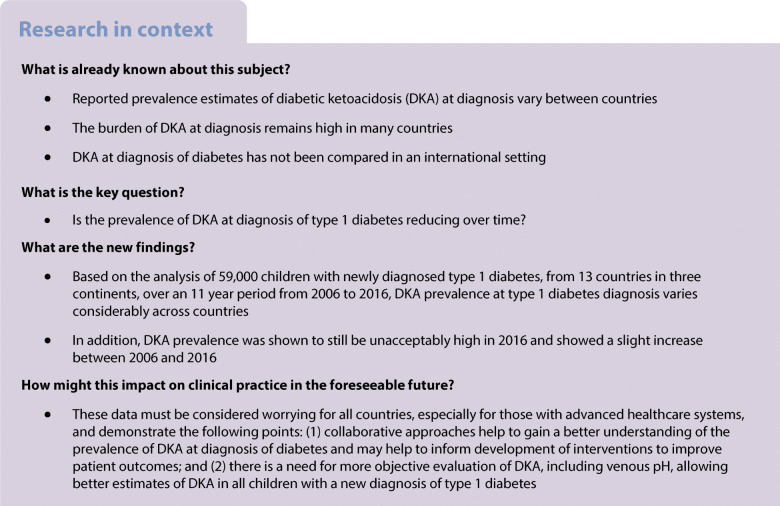



## Introduction

Clinical presentation of type 1 diabetes is often associated with diabetic ketoacidosis (DKA), which may lead to increased morbidity and mortality risk and healthcare expenditure [1]. DKA at diabetes diagnosis may arise from delayed diagnosis due to lack of awareness of diabetes symptoms or failure of a healthcare provider to consider diabetes when a child presents with abdominal pain, nausea and vomiting [2]. It is associated with both long-term poor glycaemic control and subsequent recurrent episodes of DKA [3–6]. DKA at diabetes diagnosis is also associated with long-term spatial memory performance and is associated with lower cognitive scores and altered brain growth [7, 8].

Screening of genetic risk and beta cell autoantibodies in high-risk individuals allows an early diagnosis of type 1 diabetes [9–12] and, consequently, possible prevention of DKA at diabetes diagnosis. However, a cost–benefit analysis of a screening programme in the general population showed that costs far outweighed the economic benefits [13]. The impact of individual knowledge and community-based information and education campaigns in reducing DKA at type 1 diabetes onset has been disappointing [14–16]. These studies have suggested that knowledge of the symptoms of diabetes does not necessarily appear to translate to an earlier diagnosis. It is worthwhile noting that even, in the T1D Exchange registry [17], a relatively high percentage of children who had a parent with type 1 diabetes (and, hence, knowledge of the classic symptoms of diabetes) presented with DKA (24%). Community education programmes, in which DKA was reduced when compared with a no-intervention group, were intensive and also specifically targeted primary care physicians, teachers and early childhood workers, using face-to-face education and written action plans [18, 19]. On the other hand, other studies identified clinical and sociodemographic factors associated with the presence of DKA at diabetes onset [20, 21], suggesting specific targets for intervention to raise awareness of the disease.

Reported prevalence estimates of DKA at diabetes diagnosis vary between countries and there is some evidence that prevalence is associated with a country’s socioeconomic factors [22]. However, the definitions of DKA, reporting of venous pH in medical records, inclusion criteria, age ranges of individuals included and statistical analyses differ considerably between studies [22].

Despite improvements in medical care over the years, and in diabetes care after diabetes onset, the burden of DKA at diagnosis remains high in many countries [14, 23–25]. DKA in individuals with known diabetes has been compared in an international setting, but DKA prevalence at diagnosis of type 1 diabetes has not [26]. Therefore, the aim of this study was to evaluate worldwide geographical variability and time trends in the prevalence of DKA at diagnosis of childhood-onset type 1 diabetes during 2006–2016, in developed healthcare systems, according to country, sex, age and ethnic minority status.

## Methods

### Study population

An international collaboration proposed a retrospective, population-based study on DKA at type 1 diabetes diagnosis during 2006–2016, in children aged 6 months to 14.9 years. This upper age limit was chosen since most registries for type 1 diabetes include children under 15 years of age. Children under 6 months of age were not included to exclude cases of neonatal diabetes. Requirements for participating countries included nationwide or regional availability of data for at least 6 consecutive years in the study period, and willingness to share anonymised patient-level data for joint analysis.

DKA was defined according to the International Society for Pediatric and Adolescent Diabetes (ISPAD) criteria (venous pH <7.3 or serum bicarbonate <15 mmol/l) or documented information on DKA (yes/no) according to the physician providing medical care at diagnosis.

### Data collection

Required data per individual included were age, date of diabetes diagnosis, sex, ethnic minority status (if available), and venous pH, serum bicarbonate, or information on DKA (yes/no).

**Fig. 1:**
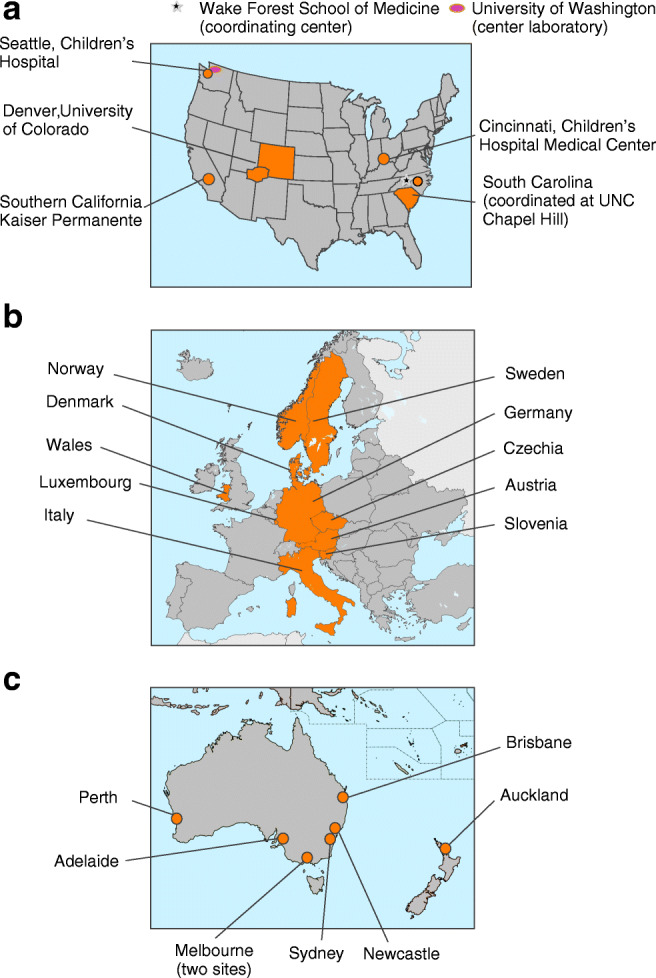
Map showing 13 centres from three continents (**a**) the USA, (**b**) Europe and (**c**) Australasia, participating in the DKA international collaboration project (whole nations, administrative units or clinic centres are shown). Maps by https://vemaps.com/, adapted by the authors

Thirteen countries participated in the project (Fig. 1): ten countries from Europe (Austria, Germany and Luxembourg [Diabetes Patients Follow-up registry (DPV)], Czechia [Czech National Childhood Diabetes Register (ČENDA)], Denmark [Danish Registry of Childhood and Adolescent Diabetes (DanDiabKids)], Italy [Study Group for diabetes of the Italian Society for Paediatric Endocrinology and Diabetes], Norway [Norwegian Childhood Diabetes Registry (NCDR)], Slovenia [Slovenian national diabetes type 1 registry], Sweden [The Swedish Paediatric Diabetes Quality Registry, SWEDIABKIDS] and the UK [Wales; The Brecon Group Register and Brecon Cohort (Welsh Paediatric Diabetes Interest Group)]); two regions from Australasia (Auckland, New Zealand and Australia [Australasian Diabetes Data Network (ADDN)]); and a consortium of sites from the USA (SEARCH for Diabetes in Youth-US).

The European participating centres defined ethnic minority status as at least one parent born outside the country with a positive migration history, or using a list of ethnic categories (African, Asian, mixed). The USA (SEARCH) included in ethnic minority status all races/ethnicities other than non-Hispanic white. The New Zealand (Auckland) centre defined ethnic minority status as Māori, Pacific Islander, Indian, South-East Asian, African or Middle Eastern ethnicities. The Australian centres included in ethnic minority status, Aboriginal and Torres Strait Islander people and immigrants from a non-Anglo background.

### Data sources

Data sources consisted of population-based registries (Australia, New Zealand [Auckland], Austria, Czechia, Denmark, Germany, Luxembourg, Norway, Slovenia, Sweden, Wales), nationwide clinical records (Italy) and a national multicentre study (USA [SEARCH]) with a completeness of coverage of at least 90% during the study period, as previously described [21, 25, 27–33]. All data owners gave the permission for publication. Data were collected from each country and integrated in a joint SAS database.

To evaluate the association of DKA prevalence with gross domestic product (GDP) per capita and human development index (HDI), the mean GDP and HDI for the 13 participating countries were extracted for 2006–2016 from the reports of the United Nations Development Programme [34]. HDI measures the development of a country using three dimensions, namely life expectancy at birth, expected years of schooling and the gross national income per capita. The association between DKA prevalence and countries’ latitudes was also analysed.

### Statistical analysis

DKA at diagnosis of type 1 diabetes was evaluated as raw and standardised prevalence and 95% CI. The prevalence of DKA was standardised by sex and age over the whole study population using the direct method. Raw and standardised prevalences were computed across the entire period, overall and by country.

Mean prevalence was also calculated according to age group and sex. Comparisons between sexes in each age group were performed using the χ^2^ test. The *p* values were adjusted for multiple comparisons using the procedure proposed by Benjamini and Hochberg [35].

DKA prevalence by country and year at diagnosis of type 1 diabetes, adjusted for sex and age groups, was estimated using multiple log–binomial regression analysis.

Unadjusted temporal trends in DKA prevalence were estimated using log–binomial regression analysis by country. Next, to explore whether changes in demographics of type 1 diabetes explain the observed trends, we performed multiple log–binomial regression analysis to estimate temporal trends adjusted for sex, age group and ethnic minority status, obtaining prevalence ratios for annual changes across countries.

The association between prevalence of DKA at diagnosis of diabetes and GDP per capita, HDI and countries’ latitude was evaluated by Spearman correlation coefficients, weighted by the number of cases of type 1 diabetes in each country and 95%CI.

All the statistical analyses were performed using the SAS System vs 9.4 (SAS Institute, Cary, NC, USA). A *p* value <0.05 was defined as statistically significant.

## Results

### DKA prevalence at type 1 diabetes diagnosis

During the 11 year period, in our cohort, there were 59,000 new diagnoses of type 1 diabetes (median age 9.0 years, interquartile range 5.5–11.7 years). Characteristics of participants are reported in electronic supplementary material (ESM) Table 1; 52.9% were male and a total of 17,205 (29.2%; 95% CI 28.82, 29.56) children were considered to have had DKA at onset of type 1 diabetes. Table 1 shows the number of children with newly diagnosed type 1 diabetes and raw and standardised DKA prevalence (95% CI). The overall mean prevalence of DKA at diagnosis was 29.9%, with six countries (Australia, Denmark, Germany, Norway, Sweden, Wales) reporting significantly lower prevalence of DKA and five countries (Austria, Italy, Luxembourg, Slovenia, USA) significantly higher prevalences. The lowest prevalence was found in Sweden, whereas the highest was observed in Luxembourg. The standardised values were very close to the raw values in all countries, showing a small effect of population structure on DKA prevalence.

**Table 1:** Cases of type 1 diabetes, DKA prevalence and standardised DKA prevalence, at type 1 diabetes diagnosis across the study population between 2006 and 2016

Country	New diagnosis of type 1 diabetes (*n*)	DKA prevalence, % (95% CI)	Standardised DKA prevalence, % (95% CI)^a^
Australia (ADDN)	4428	24.9 (23.6, 26.2)	24.9 (23.4, 26.4)
Austria	1504	38.0 (35.6, 40.5)	37.7 (34.6, 40.7)
Czechia	2261	28.8 (27.0, 30.7)	28.6 (26.4, 30.8)
Denmark	3084	20.7 (19.3, 22.1)	20.8 (19.1, 22.4)
Germany	19,127	26.8 (26.2, 27.4)	26.8 (26.1, 27.5)
Italy	10,317	41.2 (40.3, 42.2)	41.2 (39.9, 42.4)
Luxembourg	192	43.8 (36.9, 50.9)	43.8 (34.5, 53.2)
New Zealand (Auckland)	670	26.3 (23.1, 29.7)	26.3 (22.4, 30.2)
Norway	3331	22.1 (20.7, 23.5)	22.1 (20.5, 23.7)
Slovenia	471	40.3 (36.0, 44.8)	39.9 (34.2, 45.6)
Sweden	6457	19.5 (18.6, 20.5)	19.5 (18.4, 20.6)
USA (SEARCH)	5485	36.9 (35.6, 38.1)	37.0 (35.4, 38.6)
UK (Wales)	1673	25.0 (23.0, 27.2)	25.0 (22.6, 27.4)
All countries combined	59,000	29.2 (28.8, 29.6)	

^a^Standardised on whole study population

ESM Table 2 reports prevalence of DKA and the annual percentage change according to the degree of acidosis for each country, year and for all countries combined. Both moderate DKA and severe DKA overall prevalences showed a small but significant increase over the study period (1.8% and 1.6%, respectively). A high variability between countries across the study period was found both for moderate and severe forms of DKA prevalence. Germany and the USA (SEARCH) reported increasing annual percentages in moderate DKA and severe DKA prevalence; Slovenia and Luxembourg reported increasing annual percentages in the moderate and the severe forms, respectively*.*

### Sex and age differences

A significantly higher prevalence of DKA was found in girls in Denmark and Slovenia, whereas a higher prevalence was found in boys in Wales (ESM Fig. 1).

Significant differences in mean prevalence of DKA according to age group were found for all countries, except Slovenia; prevalence was higher in children under 5 years of age apart from in Sweden, Denmark and Auckland (New Zealand) where the highest proportions were found in adolescents aged 10–14.9 years (ESM Fig. 2). The overall highest mean prevalence of DKA (34.8% [95% CI 34.0, 35.6]) was found in children aged 0.5–4.9 years, whereas higher proportions were reported in adolescents than in children aged 5–9.9 years (29.6% [95% CI 29.0, 30.2] vs 25.5% [95% CI 24.9, 26.1], respectively).

**Fig. 2:**
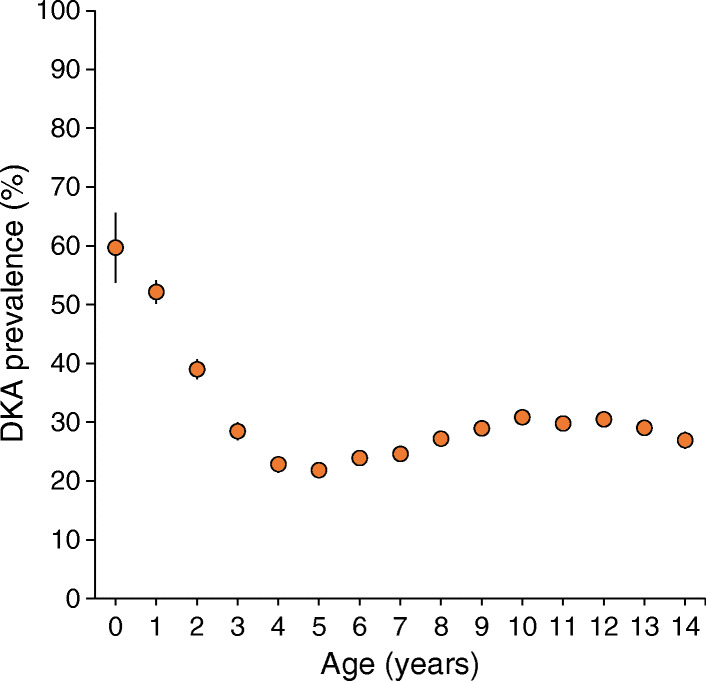
Mean DKA prevalence (per 100 people) and 95% CI at type 1 diabetes diagnosis according to year of age (all countries combined)

As shown in Fig. 2, the overall highest prevalence was observed in children under 1 year of age (59.7% [95% CI 53.7, 65.7]), with a decrease in proportions up until 5 years of age (21.9% [95% CI 20.5, 23.2]) and a slow increase thereafter, as children got older, peaking at around 10–12 years of age.

Sex differences were also found by age group (Table 2). No significant differences between boys and girls were found in the 0.5–4.9 year age group whereas higher prevalences in girls in the 5–9.9 year age group in Germany, Norway and Slovenia were reported. A significantly higher prevalence was found in Wales in boys aged 5–9.9 years and in the USA in boys aged 10–14.9 years.

**Table 2:** Mean DKA prevalence (per 100 people) at type 1 diabetes diagnosis across the study population, according to sex and age group

Country	Female sex		Male sex		*p* value^a^
	Type 1 diabetes (*n*)	DKA prevalence (95% CI)	Type 1 diabetes (*n*)	DKA prevalence (95% CI)	
0.5–4.9 years					
Australia (ADDN)	483	31.3 (26.3, 36.2)	561	32.6 (27.9, 37.3)	0.874
Austria	164	45.7 (35.4, 56.1)	220	45.0 (36.2, 53.8)	0.935
Czechia	275	34.5 (27.6, 41.5)	316	34.5 (28.0, 41.0)	0.989
Denmark	241	24.5 (18.2, 30.7)	287	17.4 (12.6, 22.2)	0.153
Germany	1948	33.0 (30.5, 35.6)	2047	31.0 (28.6, 33.4)	0.450
Italy	1115	51.0 (46.8, 55.2)	1222	50.2 (46.2, 54.1)	0.874
Luxembourg	13	76.9 (46.2, 95.5)	24	54.2 (24.8, 83.5)	0.450
New Zealand (Auckland)	56	28.6 (14.6, 42.6)	63	22.2 (10.6, 33.8)	0.694
Norway	288	24.3 (18.6, 30.0)	345	26.7 (21.2, 32.1)	0.747
Slovenia	60	53.3 (34.9, 71.8)	71	35.2 (21.4, 49.0)	0.145
Sweden	669	19.7 (16.4, 23.1)	755	17.2 (14.3, 20.2)	0.481
USA (SEARCH)	472	41.5 (35.7, 47.3)	568	45.6 (40.1, 51.1)	0.457
UK (Wales)	147	27.9 (19.4, 36.4)	169	38.5 (29.1, 47.8)	0.153
All countries combined	5931	35.2 (33.7, 36.7)	6648	34.4 (33.0, 35.8)	0.326
5–9.9 years					
Australia (ADDN)	931	24.0 (20.8, 27.1)	888	18.9 (16.1, 21.8)	0.058
Austria	247	30.8 (23.9, 37.7)	275	32.0 (25.3, 38.7)	0.874
Czechia	433	25.9 (21.1, 30.7)	453	25.4 (20.8, 30.0)	0.935
Denmark	550	20.9 (17.1, 24.7)	537	15.6 (12.3, 19.0)	0.107
Germany	3528	25.4 (23.7, 27.0)	3570	21.8 (20.3, 23.4)	0.019
Italy	1937	37.2 (34.5, 39.9)	2009	35.6 (33.0, 38.2)	0.561
Luxembourg	33	54.5 (29.4, 79.7)	35	31.4 (12.9, 50.0)	0.162
New Zealand (Auckland)	119	22.7 (14.1, 31.2)	111	18.9 (10.8, 27.0)	0.747
Norway	591	21.5 (17.8, 25.2)	620	14.8 (11.8, 17.9)	0.030
Slovenia	82	48.8 (33.7, 63.9)	91	25.3 (15.0, 35.6)	0.026
Sweden	1154	14.9 (12.7, 17.1)	1178	13.8 (11.6, 15.9)	0.694
USA (SEARCH)	1056	37.2 (33.5, 40.9)	985	32.2 (28.6, 35.7)	0.083
UK (Wales)	318	17.3 (12.7, 21.9)	262	26.7 (20.5, 33.0)	0.047
All countries combined	10,979	27.1 (26.1, 28.1)	11,014	24 (23.1, 24.9)	<0.001
10–14.9 years					
Australia (ADDN)	753	22.8 (19.4, 26.3)	812	25.1 (21.7, 28.6)	0.561
Austria	255	44.7 (36.5, 52.9)	343	35.0 (28.7, 41.2)	0.083
Czechia	347	27.4 (21.9, 32.9)	437	28.6 (23.6, 33.6)	0.874
Denmark	689	22.9 (19.4, 26.5)	780	21.9 (18.6, 25.2)	0.874
Germany	3510	26.5 (24.8, 28.2)	4524	27.5 (26.0, 29.0)	0.561
Italy	1775	41.5 (38.5, 44.5)	2259	39.6 (37.0, 42.2)	0.481
Luxembourg	32	34.4 (14.1, 54.7)	55	38.2 (21.9, 54.5)	0.874
New Zealand (Auckland)	152	32.9 (23.8, 42.0)	169	28.4 (20.4, 36.4)	0.679
Norway	645	23.9 (20.1, 27.6)	842	23.8 (20.5, 27.0)	0.981
Slovenia	82	42.7 (28.6, 56.8)	85	41.2 (27.6, 54.8)	0.935
Sweden	1183	25.0 (22.2, 27.9)	1518	24.3 (21.8, 26.8)	0.874
USA (SEARCH)	1083	32.4 (29.0, 35.8)	1321	38.2 (34.9, 41.6)	0.030
UK (Wales)	363	23.7 (18.7, 28.7)	414	24.6 (19.9, 29.4)	0.874
All countries combined	10,869	29.3 (28.3, 30.3)	13,559	29.8 (28.9, 30.7)	0.444

^a^*p* value is adjusted for multiple testing using the Benjamini–Hochberg procedure and it compares DKA prevalence in female sex vs male sex

### Ethnic minority status

The overall mean prevalence of DKA was higher in children of ethnic minority status (36.9% [95% CI 35.8, 38.0], *p*< 0.01) than in those of non-minority status (28.4% [95% CI 28.0, 28.8]) and this observation was confirmed in most countries, (ESM Fig. 3). No information on ethnic minority status was collected in Czechia and there were no children from an ethnic minority background in Slovenia.

### Temporal trend analysis

**Fig. 3:**
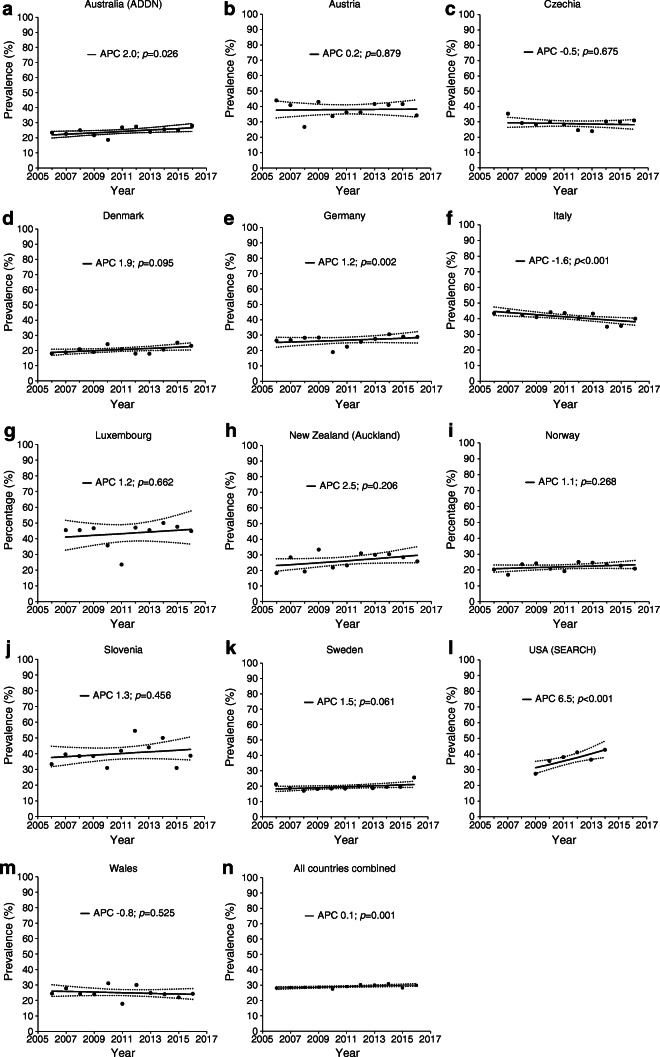
Annual DKA prevalence (per 100 people) at type 1 diabetes diagnosis and estimated annual percentage change (APC) for each country and all countries combined, during 2006–2016. Solid line, estimated prevalence trend; dashed line, 95% CI of estimated prevalence trend

Figure 3 reports the unadjusted annual percentage change in DKA prevalence at type 1 diabetes diagnosis and ESM Fig. 4 the estimated annual standardised prevalence of DKA by country. A slight increasing temporal trend of 0.7% per year over the period of 11 years for all countries combined was observed. Table 3 shows the results of multiple log–binomial regression analysis. A significant increase in prevalence of DKA at diagnosis of diabetes was found in Australia, Germany and USA, whereas a significant decrease was observed in Italy during the study period.

**Table 3:** Time trend for DKA prevalence at type 1 diabetes diagnosis, adjusted for sex, age group and ethnic minority status

Country	Year at diabetes diagnosis	Female vs male sex	Age < 5 years vs 10–14.9 years	Age 5–9.9 years vs 10–14.9 years	Ethnic minority vs non-minority status
Australia (ADDN)	1.024 (1.002, 1.046)	1.014 (0.893, 1.152)	1.420 (1.214, 1.660)	0.930 (0.795, 1.089)	1.123 (0.980, 1.286)
Austria	1.000 (0.979, 1.021)	1.105 (0.974, 1.255)	1.157 (0.999, 1.339)	0.789 (0.673, 0.927)	1.361 (1.188, 1.558)
Czechia^a,b^	0.995 (0.972, 1.020)	0.993 (0.872, 1.130)	1.227 (1.048, 1.438)	0.911 (0.777, 1.068)	–
Denmark	1.021 (0.999, 1.044)	1.179 (1.027, 1.353)	0.911 (0.751, 1.103)	0.809 (0.692, 0.947)	1.533 (1.255, 1.874)
Germany	1.011 (1.003, 1.018)	1.048 (1.000, 1.098)	1.178 (1.112, 1.248)	0.868 (0.822, 0.918)	1.281 (1.208, 1.358)
Italy	0.988 (0.980, 0.995)	1.039 (0.992, 1.088)	1.244 (1.177, 1.315)	0.895 (0.846, 0.947)	1.170 (1.085, 1.262)
Luxembourg	1.000 (0.944, 1.059)	1.312 (0.968, 1.777)	1.743 (1.203, 2.525)	1.152 (0.783, 1.695)	1.075 (0.774, 1.493)
New Zealand (Auckland)	1.021 (0.982, 1.061)	1.187 (0.921, 1.530)	0.827 (0.583, 1.173)	0.683 (0.505, 0.923)	0.881 (0.652, 1.192)
Norway	1.010 (0.990, 1.031)	1.077 (0.949, 1.223)	1.055 (0.898, 1.239)	0.751 (0.646, 0.872)	1.538 (1.262, 1.875)
Slovenia^a,b^	1.004 (0.971, 1.037)	1.411 (1.126, 1.768)	1.076 (0.830, 1.396)	0.906 (0.696, 1.179)	–
Sweden	1.015 (0.999, 1.031)	1.057 (0.958, 1.166)	0.757 (0.666, 0.86)	0.582 (0.516, 0.655)	1.567 (1.255, 1.958)
USA (SEARCH)	1.063 (1.042, 1.085)	0.954 (0.891, 1.022)	1.234 (1.132, 1.346)	0.986 (0.911, 1.068)	1.141 (1.063, 1.224)
UK (Wales)	0.993 (0.967, 1.020)	0.785 (0.664, 0.929)	1.368 (1.121, 1.670)	0.904 (0.741, 1.105)	1.280 (0.933, 1.756)
All countries combined^a^	1.007 (1.003, 1.011)	1.038 (1.012, 1.064)	1.176 (1.140, 1.212)	0.863 (0.838, 0.889)	–

Data are presented as prevalence ratio (95% CI)

^a^Adjusted for sex and age group only

^b^Information on ethnic minority status not given for this country

Girls were at a significantly higher risk of DKA at diagnosis in Denmark, Germany and Slovenia.

A significant negative association between adjusted DKA prevalence and HDI (*r* = −0.75 [95% CI −0.90, −0.42]) and latitude (*r* = −0.55 [95%CI −0.81, −0.10]) was also observed, whereas no significant association was found with GDP per capita (*r* = −0.47 [95% CI −0.77, 0.01]) (ESM Fig. 5).

## Discussion

The major findings of this international joint project were a high prevalence of children presenting with DKA at diagnosis and a slight increasing trend in prevalence of DKA at diagnosis of type 1 diabetes during 2006–2016 across sites. The project also highlights large inter-country differences and variability in the prevalence of DKA at diabetes diagnosis by age group, sex and ethnic minority status. During the 11 years studied, a marked increase in DKA at diabetes diagnosis was clear in the USA (SEARCH) and Australia (ADDN), while in Germany the increase was small but statistically significant due to the large number of patients from this country. In particular, increasing burden of DKA among both youth [36] and adults [37] was also reported in recent years. The proportion of children presenting with DKA at diagnosis of diabetes decreased in Italy over the study years. However, Italy showed one of the highest prevalences of DKA. The overall increase in DKA prevalence, although small, is indicative of a general phenomenon that remains constantly high over time and shows no sign of decreasing.

Several studies indicate that DKA is associated with a delay in diagnosis [2, 38] and many children presenting with DKA have had a medical encounter before diagnosis [2]. Increased disease awareness allows for an earlier diagnosis of type 1 diabetes, likely preventing the occurrence of DKA, as suggested by the lower proportion of children diagnosed with DKA in countries where the incidence of type 1 diabetes is higher [24, 38, 39]. In addition, screening programmes for diabetes [11, 40, 41] as well as publicity campaigns [18] have been considered effective in reducing DKA prevalence at type 1 diabetes diagnosis. However, awareness campaigns targeted to prevent DKA at type 1 diabetes diagnosis have not shown uniform results. A reduction in DKA prevalence was reported in Italy [18] and Australia [19], whereas no changes were found in Austria [14] and Wales [30].

Moreover, as recently reviewed [42], all the above-mentioned studies are limited by methodological issues related to their observational designs. However, most studies showed that an awareness campaign targeted to large-scale populations, and interventions targeted to alert primary healthcare to the symptoms of type 1 diabetes, are both feasible and acceptable. Our study did not collect information on local awareness campaigns but showed an increasing trend in the prevalence of DKA at diagnosis of type 1 diabetes over the 11 year period, suggesting that if prevention campaigns had been carried out locally, the expected effect was not achieved. Effective prevention campaigns should be targeted to reach a high percentage of the general population and healthcare professionals, should be repeated and prolonged over time, and should monitor all factors influencing the occurrence of DKA [15]. Preschool screening for islet autoantibodies in the general population might be useful to prevent DKA at diagnosis, as shown in a public health screening programme among children aged 2–5 years in Bavaria, Germany, in which the prevalence of DKA at diagnosis was less than 5% [43]. Moreover, screening costs were kept low by using a relatively inexpensive and sensitive method.

### Differences in DKA prevalence between countries

The proportion of children with DKA at diagnosis of type 1 diabetes varies widely between countries. In Europe, the North–South gradient was confirmed by a higher prevalence of DKA in countries with low incidence of type 1 diabetes and in those closer to the equator, compared with countries having a higher incidence of type 1 diabetes and those that are further from the equator [22, 44]. In addition, higher proportions of DKA are more common in countries with a lower HDI. Our data are consistent with these results, showing an inverse association between DKA prevalence and HDI and geographical latitude.

### Sex differences in DKA prevalence

In our analysis, the overall proportion of girls presenting with DKA was higher than among boys. Almost all countries showed a slightly higher proportion of girls presenting with DKA at diabetes diagnosis, with the exception of Wales where boys more often presented with DKA at diabetes diagnosis. The reason for the higher incidence of DKA in the male sex [44] but a higher proportion of DKA in the female sex is unclear and calls for further studies. Even if an excess of DKA occurs in girls, as was recently reported in a large series of children with established type 1 diabetes [26], a plausible biological, physiological or clinical explanation is currently very hard to hypothesise. Perhaps girls are more reluctant to see the associated weight loss as problematic and, therefore, do not recognise early symptoms of DKA.

### Age differences in DKA prevalence

The analysis of this multicentre large dataset confirms the highest risks of DKA occur in younger children in all countries except Sweden, Denmark and New Zealand (Auckland), where the highest prevalence of DKA were registered in adolescents aged 10–14.9 years. These findings could be due to less parental awareness of symptoms in very young age groups, or the reluctance of adolescents to bring their symptoms to parental attention. Our results, showing a very high prevalence in children under 3 years of age, are consistent with previous observation of higher DKA risk among the youngest children [21, 25, 39, 45], in whom it may be particularly difficult to recognise signs and symptoms, leading to delayed diagnosis. Moreover, it has been reported that in very young children there is more extensive beta cell destruction at diabetes onset [46], which could be related to a faster and more aggressive disease onset.

### Differences in DKA prevalence according to ethnic minority status

In most countries included in our study, an ethnic minority status was associated with a higher prevalence of DKA at diabetes diagnosis, as reported in previous worldwide studies [21, 39]. An ethnic minority status might act as a social and cultural barrier, negatively affecting the awareness of disease, the early recognition of symptoms, and the equity in accessing healthcare services.

### Strengths and limitations

Strengths of our study include worldwide collaboration, which allowed the collection of data from a large number of cases of type 1 diabetes. Moreover, most of the data used for this study came from population-based registries or clinical registries with well-defined reference populations. In addition, data collection was based on standard inclusion criteria and consistent definitions of DKA over time. Most countries had data on venous pH and/or serum bicarbonate levels (11 countries, including 44,617 and 22,426 cases with venous pH and bicarbonate data, respectively).

Our study has several limitations. Results were based on retrospective data collection and the study period was relatively short, limiting the precision of trend estimates in each country. We only included cases of type 1 diabetes with available information on DKA status, which was the same approach used in similar studies on DKA at diagnosis of type 1 diabetes [24]. Consequently, the exclusion of data from individuals without information on venous pH and/or bicarbonate might have caused an overestimation of DKA prevalence. However, in more than 80% of the cases, data for venous pH or bicarbonate were available. We suspect that physicians do not measure venous pH or bicarbonate values if clinical symptoms of type 1 diabetes at diagnosis are not severe or, even, absent, since guidelines concerning children [47] do not clearly indicate that measurement of venous pH and/or bicarbonate is mandatory at diagnosis of type 1 diabetes. If an individual had mild symptoms and was not suspected to have DKA (and, therefore, venous pH was not measured), that person would have been ‘physician diagnosed’ as not having DKA. Such evaluation may bias the prevalence estimates, potentially underestimating the observed burden.

While venous pH and/or bicarbonate provide reliable data on the proportions of new cases presenting with DKA, this might not be possible in all settings and potentially raises cost implications. Serum bicarbonate can be a reliable substitute for venous pH in settings where access to venous pH measurement is limited [48]. A basic metabolic profile (including plasma glucose, venous pH or bicarbonate, blood urea nitrogen, serum creatinine and serum electrolytes) and, preferably, a blood ketone measurement (or, at least, a urine measurement) should be the standard of care for evaluating new-onset diabetes mellitus in a child.

### Summary

In summary, this analysis of 59,000 children with newly diagnosed type 1 diabetes, from different countries, showed a high prevalence of children presenting with DKA at diabetes diagnosis with large inter-country differences and a slight increase in prevalence over time. Despite the present lack of effective measures to reduce the risk of DKA at diabetes diagnosis, our findings of high prevalence of DKA at diagnosis of diabetes must be considered worrying for all countries and especially for those with access to advanced healthcare systems. These findings should be considered as a call for action to put more effort into promoting an earlier diagnosis of diabetes to prevent DKA in children and adolescents worldwide. Collaborative approaches such as this help gain a better understanding of the prevalence of DKA at diagnosis of diabetes, to inform development of interventions to improve patient outcomes.

## Electronic supplementary material

ESM 1 (PDF 389 kb)

## Data Availability

The dataset for the current study is available on reasonable request by contacting RWH or VC.

## Abbreviations

ADDN: Australasian Diabetes Data Network
DKA: Diabetic ketoacidosis
GDP: Gross domestic product
HDI: Human development index
