# Supplementary material for: Temporal trends in diabetic ketoacidosis at diagnosis of paediatric type 1 diabetes between 2006 and 2016: results from 13 countries in three continents
Source: Diabetologia. 2020 May 8;63(8):1530–41. doi: 10.1007/s00125-020-05152-1 (PMC7351855; doi:10.1007/s00125-020-05152-1)
Supplement: Supplementary file 1 — (PDF 389 kb) [file 125_2020_5152_MOESM1_ESM.pdf]

ESM Table 1. Characteristics of participants

	Type 1 diabetes cases (n)	Gender (M) (%)	Age at onset median (IQR)	Minority Status %	DKA cases %	DKA cases identified by			
						pH only n (%)	serum bicarbonate only n (%)	pH or serum bicarbonate n (%)	physician documentation only n (%)
Australia (ADDN)	4428	51.1	8.3 (5.2-11.1)	29.0	24.9	0	0	0	1101 (100)
Austria	1504	55.7	8.6 (4.9-11.6)	21.4	38.0	239 (41.8)	2 (0.35)	327 (57.2)	4 (0.7)
Czechia	2261	53.3	8.1 (4.9-11.1)	-	28.7	651 (100)	0	0	0
Denmark	3084	52.0	9.7 (6.4-12.3)	8.1	21.6	15 (2.4)	621 (97.5)	1 (0.2)	0
Germany	19127	53.0	9.0 (5.6-11.9)	14.6	26.8	2890 (56.4)	64 (1.25)	1962 (38.3)	209 (4.1)
Italy	10317	53.2	8.0 (5.0-11.0)	7.7	41.2	3280 (77.2)	0	970 (22.82)	0
Luxembourg	192	59.4	8.9 (5.8-12.2)	26.6	43.8	0	3 (3.6)	81 (964)	0
New Zealand (Auckland)	670	51.2	9.5 (6.1-12.1)	24.6	26.3	1 (0.57)	0	175 (99.4)	0
Norway	3331	54.2	9.3 (5.9-12.1)	6.8	22.1	42 (5.7)	1 (0.1)	692 (94.2)	0
Slovenia	471	52.4	8.1 (4.5-11.2)	-	40.3	0	0	190 (100)	0
Sweden	6457	53.4	9.0 (5.4-11.9)	2.6	19.5	201 (15.9)	4 (0.3)	1056 (83.7)	0
USA (SEARCH)	5485	52.4	9.4 (5.9-11.9)	33.4	36.8	71 (3.5)	259 (12.8)	1034 (51.2)	657 (32.5)
Wales	1673	50.5	9.5 (6.1-12.1)	4.9	25.5	0	0	0	419 (100)
All countries combined	59000	52.9	9.0 (5.5-11.7)	13.0	29.2	7390 (42.9)	954 (5.5)	6488 (37.7)	2390 (13.9)

ESM Table 2. Prevalence of DKA according to the degree of acidosis for each country and year

Moderate DKA		Austria	Czechia	Denmark	Germany	Italy	Luxembourg	New Zealand (Auckland)	Norway	Slovenia	Sweden	USA (SEARCH)	All countries combined
2006	n	82		267	1509	806		71	261	45	647		3688
	Prevalence	32.9		10.1	16.6	24.7		11.3	9.6	24.4	10.5		16.7
	95% CI	22.5 - 43.3		6.5 - 13.8	14.7 - 18.4	21.7 - 27.7		3.7 - 18.8	6 - 13.2	11.4 - 37.5	8.1 - 12.9		15.5 - 17.9
2007	n	93	144	261	1682	801	22	60	282	43	585		3973
	Prevalence	24.7	22.2	12.3	16.6	22.8	18.2	11.7	9.6	25.6	8.4		16.3
	95% CI	15.8 - 33.7	15.4 - 29.1	8.3 - 16.3	14.8 - 18.4	19.9 - 25.8	0.7 - 35.7	3.3 - 20	6.1 - 13	12 - 39.2	6.1 - 10.6		15.1 - 17.4
2008	n	105	157	264	1700	880	11	57	297	26	548		4045
	Prevalence	15.2	12.7	13.3	18.6	23.2	36.4	12.3	14.8	3.8	9.1		17.3
	95% CI	8.2 - 22.2	7.5 - 18	9.1 - 17.4	16.8 - 20.5	20.4 - 26	2.5 - 70.3	3.5 - 21.1	10.8 - 18.9	0 - 11.8	6.7 - 11.5		16.1 - 18.4
2009	n	114	169	284	1668	1047	15	60	323	26	622	849	5177
	Prevalence	24.6	16.6	10.6	16.8	23.5	6.7	21.7	11.1	26.9	9.3	10.1	15.7
	95% CI	16.5 - 32.6	10.9 - 22.2	7 - 14.2	15 - 18.6	20.9 - 26.1	0 - 21	10.9 - 32.4	7.7 - 14.6	8.7 - 45.2	7 - 11.6	8.1 - 12.2	14.7 - 16.7
2010	n	107	207	294	1520	945	14	55	293	42	560	677	4714
	Prevalence	19.6	18.8	14.3	11.7	24.7	35.7	12.7	11.6	9.5	10.0	14.3	15.2
	95% CI	12 - 27.3	13.5 - 24.2	10.3 - 18.3	10.1 - 13.3	21.9 - 27.4	7 - 64.4	3.6 - 21.8	7.9 - 15.3	0.3 - 18.8	7.5 - 12.5	11.7 - 17	14.2 - 16.2
2011	n	155	250	277	1637	1048	17	56	291	43	593	793	5160
	Prevalence	20.6	16.8	10.5	15.1	25.5	17.6	8.9	11.3	20.9	8.8	13.2	16.0
	95% CI	14.2 - 27.1	12.1 - 21.5	6.8 - 14.1	13.4 - 16.8	22.8 - 28.1	0 - 37.9	1.2 - 16.6	7.7 - 15	8.3 - 33.6	6.5 - 11.1	10.9 - 15.6	15 - 17
2012	n	188	268	251	1913	1011	17	55	291	44	582	802	5422
	Prevalence	23.9	11.6	8.0	16.0	24.0	29.4	16.4	17.2	43.2	10.7	19.0	17.4
	95% CI	17.8 - 30.1	7.7 - 15.4	4.6 - 11.3	14.4 - 17.7	21.4 - 26.7	5.3 - 53.6	6.3 - 26.5	12.8 - 21.5	27.9 - 58.4	8.1 - 13.2	16.2 - 21.7	16.4 - 18.4
2013	n	149	267	302	1858	1026	22	60	317	50	581	850	5482
	Prevalence	24.8	13.9	9.6	17.5	26.1	31.8	15.0	14.8	28.0	10.2	16.7	17.8
	95% CI	17.8 - 9.7	9.7 - 18	6.3 - 12.9	15.8 - 19.2	23.4 - 28.8	10.7 - 53	5.7 - 24.3	10.9 - 18.8	15.1 - 40.9	7.7 - 12.6	14.2 - 19.2	16.8 - 18.8
2014	n	188	231	296	1851	915	24	56	316	48	615	857	5397
	Prevalence	28.2	18.6	12.2	20.1	22.4	33.3	17.9	12.0	39.6	9.1	18.7	18.5
	95% CI	21.7 - 13.6	13.6 - 23.7	8.4 - 15.9	18.3 - 21.9	19.7 - 25.1	13 - 53.7	7.5 - 28.2	8.4 - 15.6	25.2 - 53.9	6.8 - 11.4	16.1 - 21.3	17.5 - 19.6
2015	n	159	290	298	1857	878	21	74	339	42	589		4547
	Prevalence	29.6	17.2	14.8	20.1	23.0	23.8	16.2	14.2	19.0	8.1		18.4
	95% CI	22.4 - 12.9	12.9 - 21.6	10.7 - 18.8	18.3 - 21.9	20.2 - 25.8	3.9 - 43.7	7.6 - 24.8	10.4 - 17.9	6.7 - 31.4	5.9 - 10.4		17.3 - 19.5
2016	n	164	278	290	1932	960	29	66	321	62	535		4637
	Prevalence	23.8	16.2	14.8	20.1	24.2	37.9	10.6	10.6	32.3	13.1		19.2
	95% CI	17.2 - 11.8	11.8 - 20.5	10.7 - 18.9	18.3 - 21.9	21.5 - 26.9	19.1 - 56.7	3 - 18.2	7.2 - 14	20.3 - 44.2	10.2 - 16		18 - 20.3
APC		1.5	-0.9	1.8	2.3	0.03	6.5	1.4	1.9	5.2	1.1	11.1	1.8
p		0.349	0.642	0.25	<0.001	0.955	0.125	0.635	0.199	0.039	0.344	<0.001	<0.001

ESM Table 2. Prevalence of DKA according to the degree of acidosis for each country and year (continue)

Severe DKA		Austria	Czechia	Denmark	Germany	Italy	Luxembourg	New Zealand (Auckland)	Norway	Slovenia	Sweden	USA (SEARCH)	All countries combined
2006	n	82	0	241	1509	806		71	261	45	647		3688
	Prevalence	25.6	0.0	0.8	12.3	9.9		4.2	5.0	17.8	4.8		9.3
	95% CI	16 - 35.3	0 - 0	0 - 2	10.7 - 14	7.9 - 12		0 - 9.0	2.3 - 7.6	6.2 - 29.4	3.1 - 6.4		8.4 - 10.3
2007	n	93	144	246	1682	801	22	60	282	43	585		3973
	Prevalence	18.3	14.6	3.3	12.0	11.2	13.6	3.3	3.5	14.0	5.3		9.8
	95% CI	10.3 - 26.3	8.7 - 20.4	1 - 5.5	10.4 - 13.5	9 - 13.4	0 - 29.2	0 - 8.0	1.4 - 5.7	3.2 - 24.7	3.5 - 7.1		8.9 - 10.7
2008	n	105	157	249	1700	880	11	57	297	26	548		4045
	Prevalence	11.4	8.3	1.6	14.3	10.7	0.0	1.8	8.1	3.8	3.8		10.2
	95% CI	5.2 - 17.6	3.9 - 12.6	0 - 3.2	12.6 - 16	8.6 - 12.7		0 - 5.3	5 - 11.2	0 - 11.8	2.2 - 5.4		9.3 - 11.1
2009	n	114	169	268	1668	1047	15	60	323	26	622	849	5177
	Prevalence	15.8	7.7	2.2	10.9	11.3	6.7	8.3	5.9	23.1	4.7	1.3	7.9
	95% CI	9 - 22.6	3.6 - 11.8	0.5 - 4	9.4 - 12.4	9.4 - 13.2	0 - 21	1.1 - 15.5	3.3 - 8.5	5.7 - 40.4	3 - 6.3	0.5 - 2.1	7.1 - 8.6
2010	n	107	207	286	1520	945	14	55	293	42	560	677	4714
	Prevalence	13.1	9.7	3.8	8.4	13.3	21.4	10.9	4.8	2.4	5.5	2.4	7.8
	95% CI	6.6 - 19.6	5.6 - 13.7	1.6 - 6.1	7 - 9.7	11.2 - 15.5	0 - 46	2.4 - 19.4	2.3 - 7.2	0 - 7.2	3.6 - 7.4	1.2 - 3.5	7.1 - 8.6
2011	n	155	250	266	1637	1048	17	56	291	43	593	793	5160
	Prevalence	11.6	4.8	3.4	10.5	12.2	5.9	7.1	5.5	11.6	3.9	2.0	7.8
	95% CI	6.5 - 16.7	2.1 - 7.5	1.2 - 5.6	9 - 12	10.2 - 14.2	0 - 18.4	0.2 - 14.1	2.9 - 8.1	1.6 - 21.6	2.3 - 5.4	1 - 3	7.1 - 8.6
2012	n	188	268	240	1913	1011	17	55	291	44	582	802	5422
	Prevalence	12.8	5.2	0.8	11.1	11.3	17.6	5.5	8.2	22.7	4.6	4.9	8.7
	95% CI	8 - 17.6	2.5 - 7.9	0 - 2	9.7 - 12.5	9.3 - 13.2	0 - 37.9	0 - 11.7	5.1 - 11.4	9.8 - 35.6	2.9 - 6.4	3.4 - 6.4	8 - 9.5
2013	n	149	267	297	1858	1026	22	60	317	50	581	850	5482
	Prevalence	13.4	8.2	0.0	11.9	12.0	27.3	13.3	9.5	6.0	3.8	3.5	8.9
	95% CI	7.9 - 4.9	4.9 - 11.6		10.5 - 13.4	10 - 14	7.1 - 47.5	4.5 - 22.2	6.2 - 12.7	0 - 12.8	2.2 - 5.3	2.3 - 4.8	8.1 - 9.6
2014	n	188	231	284	1851	915	24	56	316	48	615	857	5397
	Prevalence	17.0	8.2	1.8	13.5	12.7	20.8	5.4	5.4	16.7	5.0	4.1	9.6
	95% CI	11.6 - 4.7	4.7 - 11.8	0.2 - 3.3	11.9 - 15	10.5 - 14.8	3.3 - 38.4	0 - 11.4	2.9 - 7.9	5.7 - 27.6	3.3 - 6.8	2.8 - 5.4	8.8 - 10.4
2015	n	159	290	290	1857	878	21	74	339	42	589		4547
	Prevalence	16.4	9.3	2.1	14.2	12.2	23.8	10.8	6.2	9.5	3.7		10.8
	95% CI	10.5 - 5.9	5.9 - 12.7	0.4 - 3.7	12.6 - 15.8	10 - 14.4	3.9 - 43.7	3.6 - 18.1	3.6 - 8.8	0.3 - 18.8	2.2 - 5.3		9.9 - 11.7
2016	n	164	278	278	1932	960		66	321	62	535		4637
	Prevalence	23.8	7.9	3.6	13.8	12.6		6.1	4.4	14.5	6.2		11.2
	95% CI	17.2 - 4.7	4.7 - 11.1	1.4 - 5.8	12.3 - 15.4	10.5 - 14.7		0.1 - 12	2.1 - 6.6	5.5 - 23.5	4.1 - 8.2		10.3 - 12.1
APC		-1.0	-2.5	1.1	1.5	1.7	1.7	7.4	1.3	-0.7	0.2	21.7	1.6
p		0.639	0.327	0.784	0.014	0.054	0.007	0.100	0.56	0.848	0.929	<0.001	<0.001

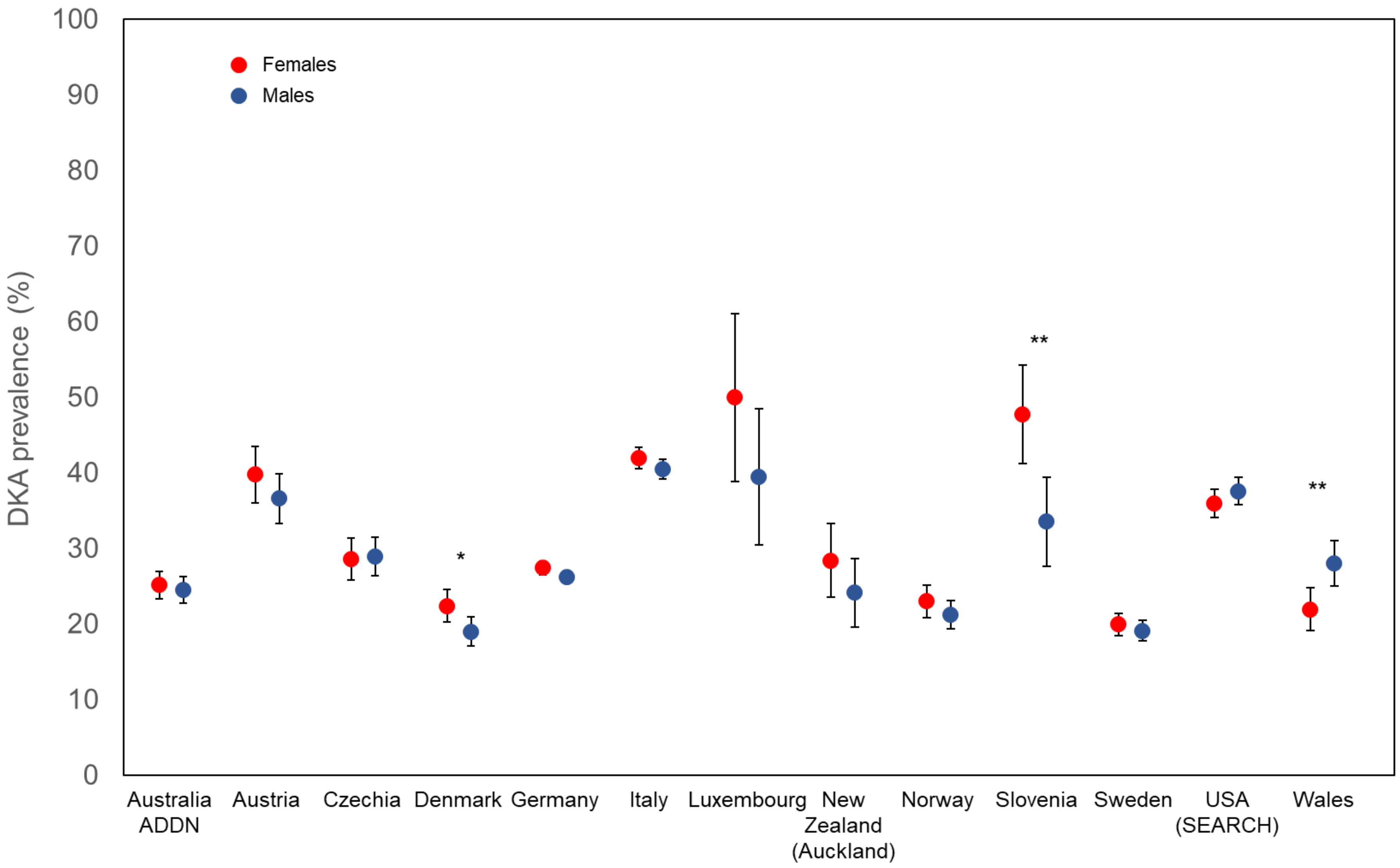

Chi-square test, * *p<0.05*; ** *p≤0.01*

ESM Figure 1. Average DKA prevalence (per 100 people) at diagnosis of type 1 diabetes and 95%CI across study population, by sex

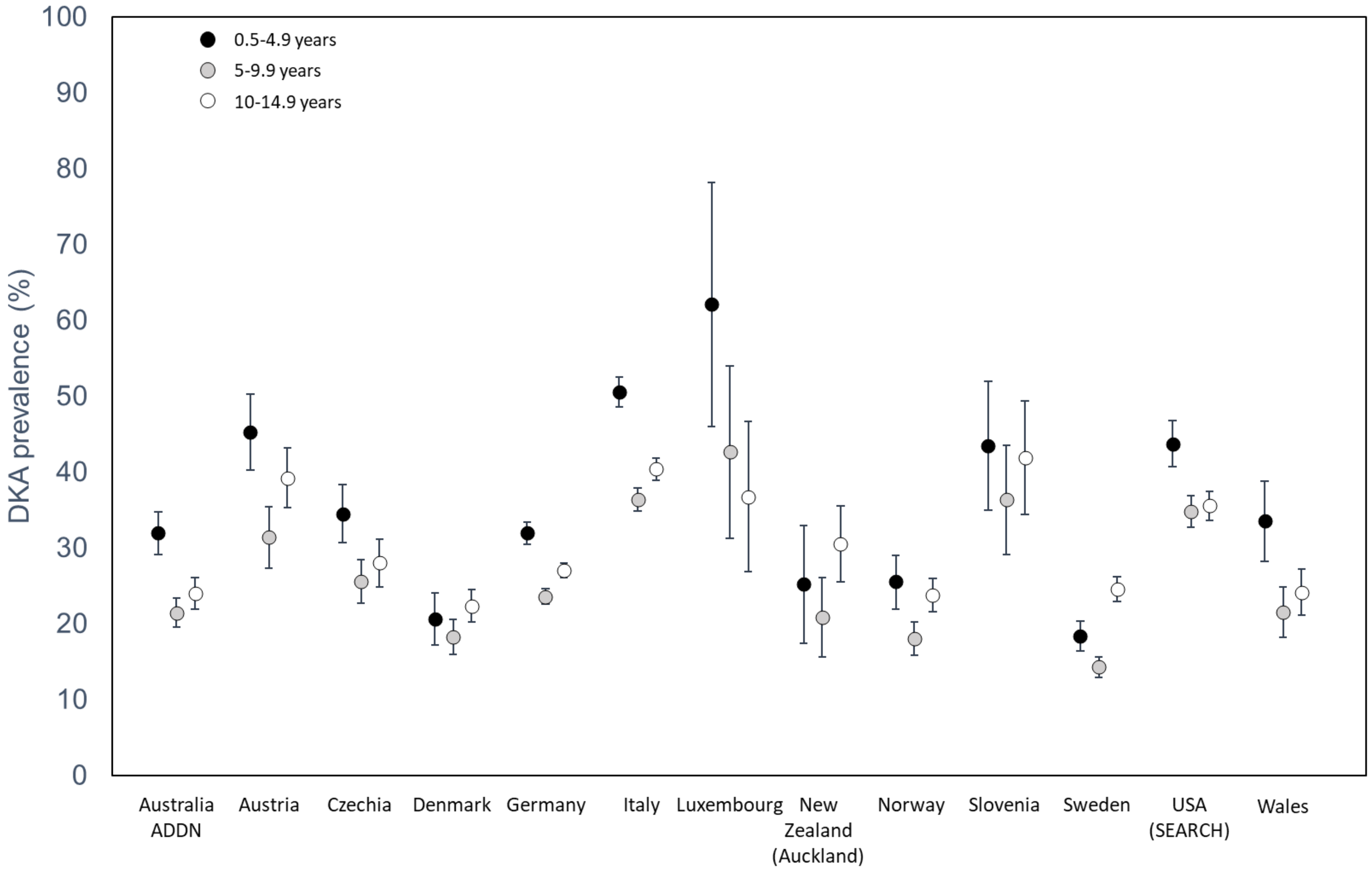

Chi-square test, * $p<0.05$; ** $p\leq0.001$

ESM Figure 2. Average DKA prevalence (per 100 people) at diagnosis of type 1 diabetes and 95%CI across study populations, according to age groups.

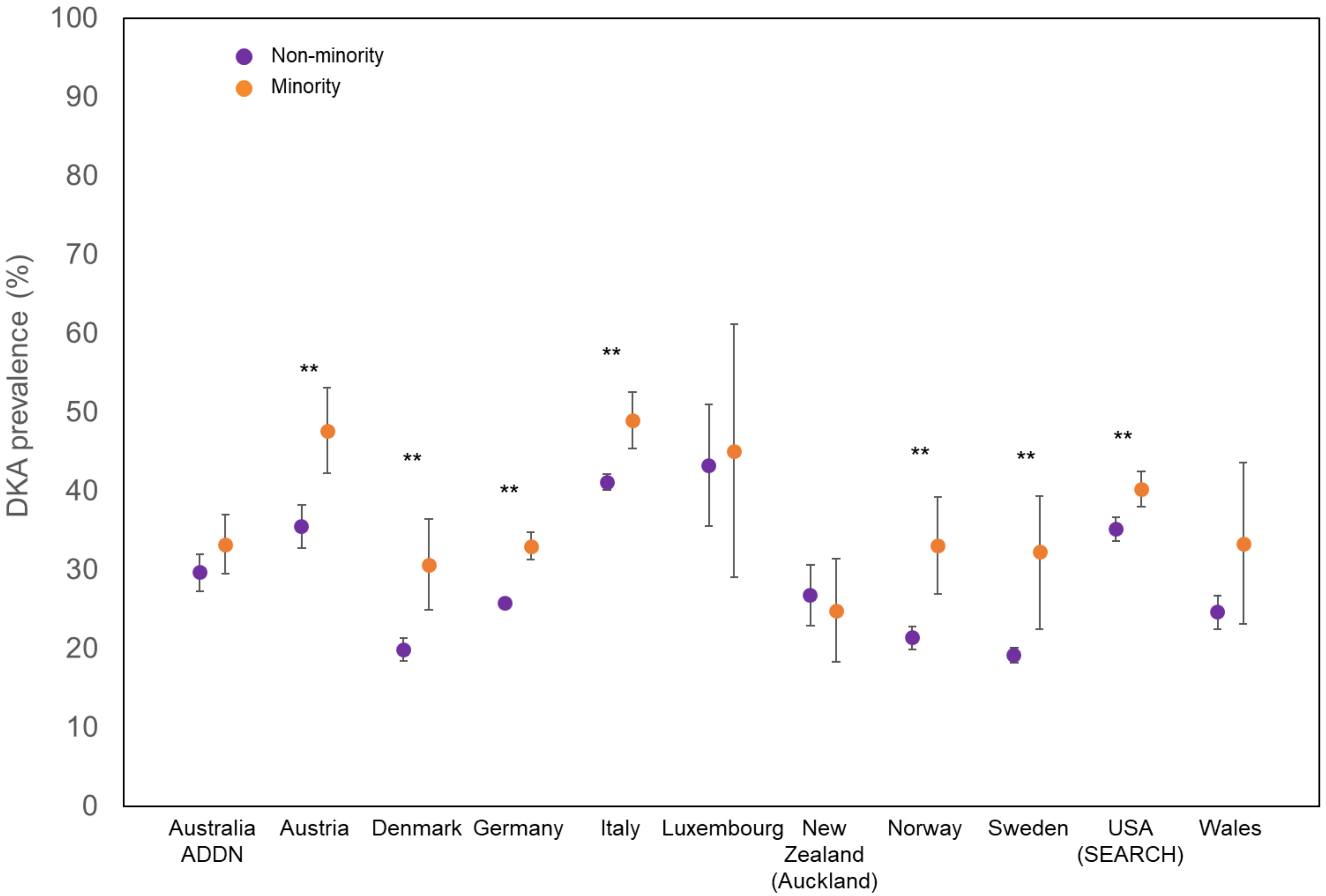

Chi-square test, ** *p<0.001*

ESM Figure 3. Average DKA prevalence (per 100 people) at diabetes of type 1 onset and 95%CI across study populations, according to minority status.

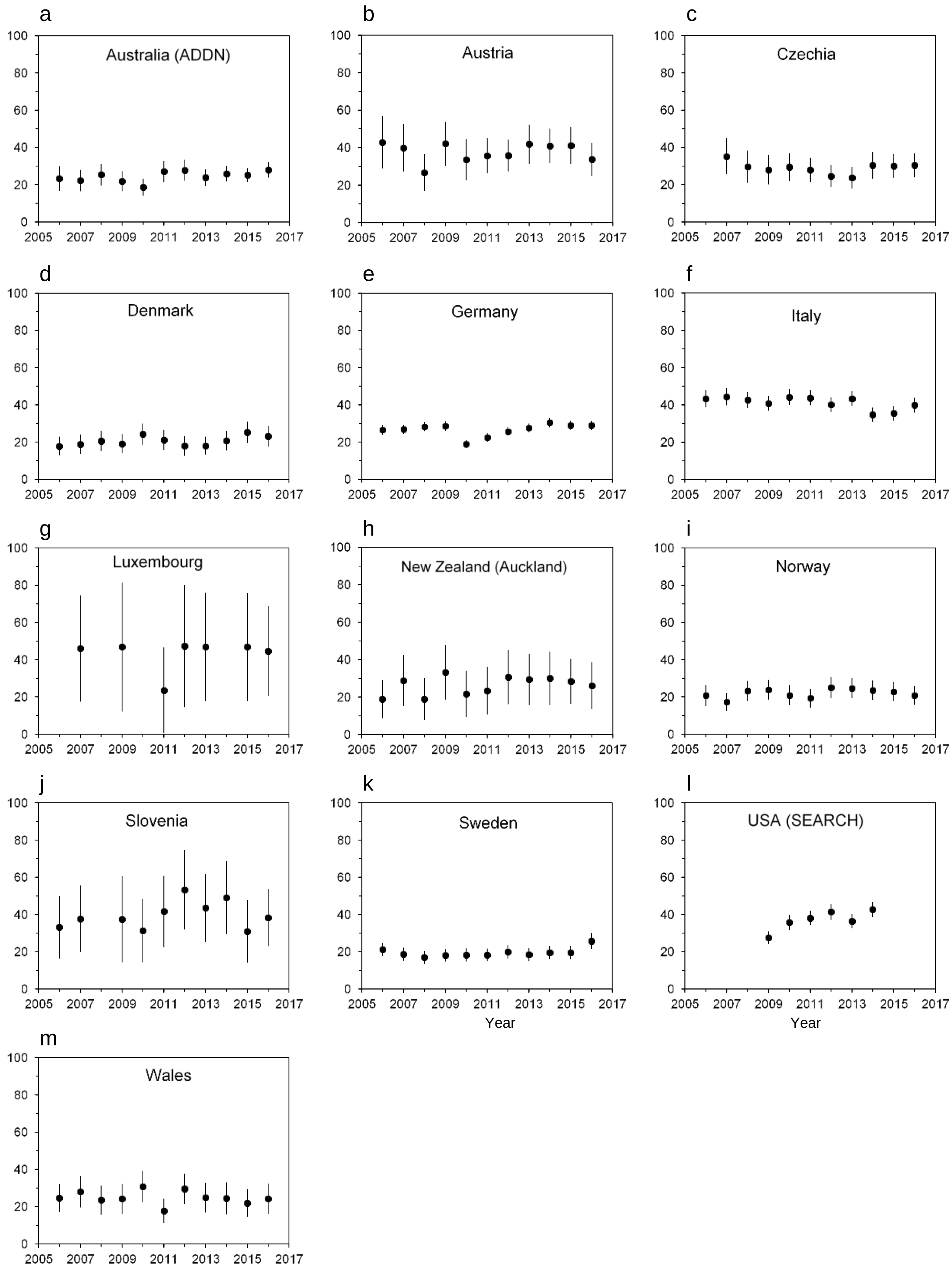

ESM Figure 4. Prevalence of DKA at diagnosis of type 1 diabetes and 95%CI per country and year adjusted for sex, age-group, and minority status (when available)

DKA prevalence for Czechia and Slovenia adjusted by sex and age-groups

Panel A

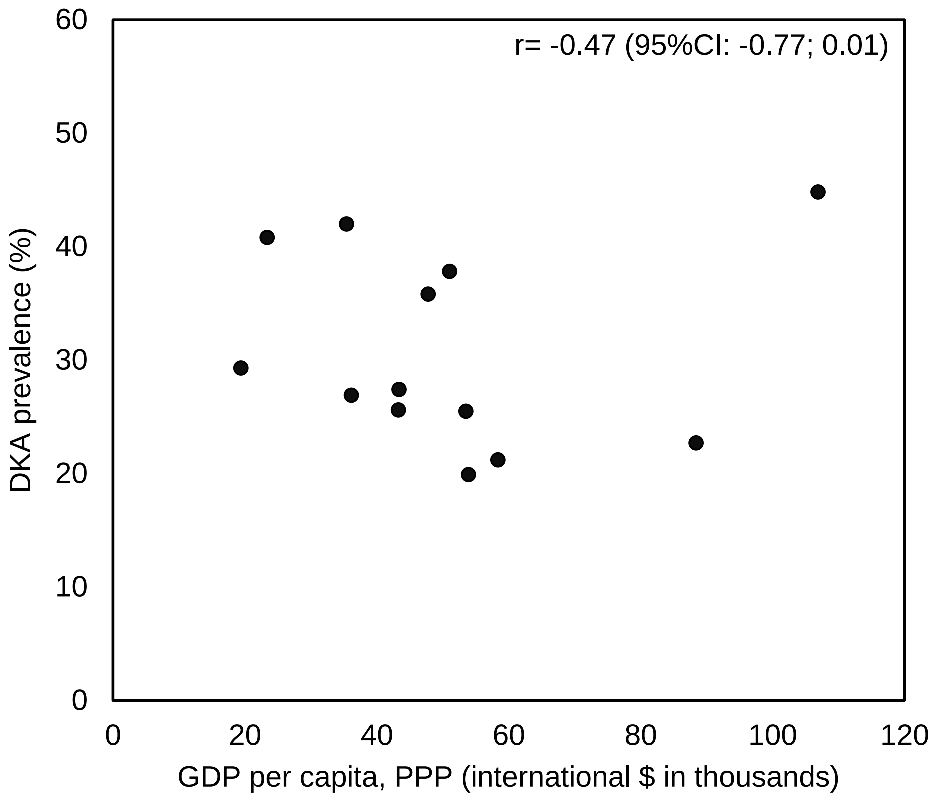

Panel B

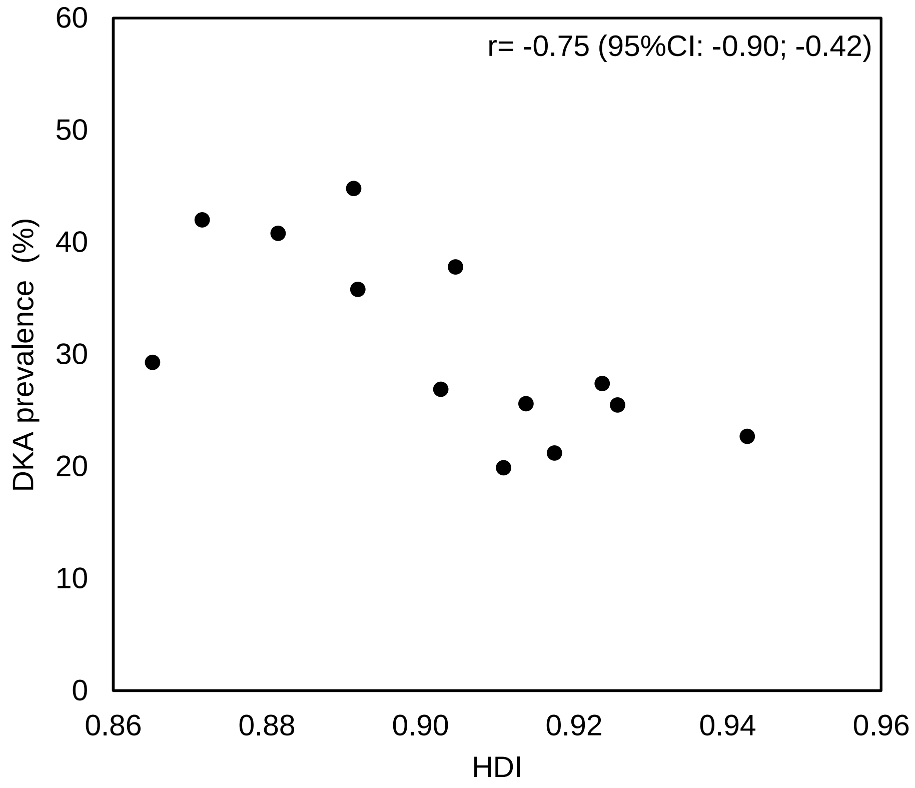

Panel C

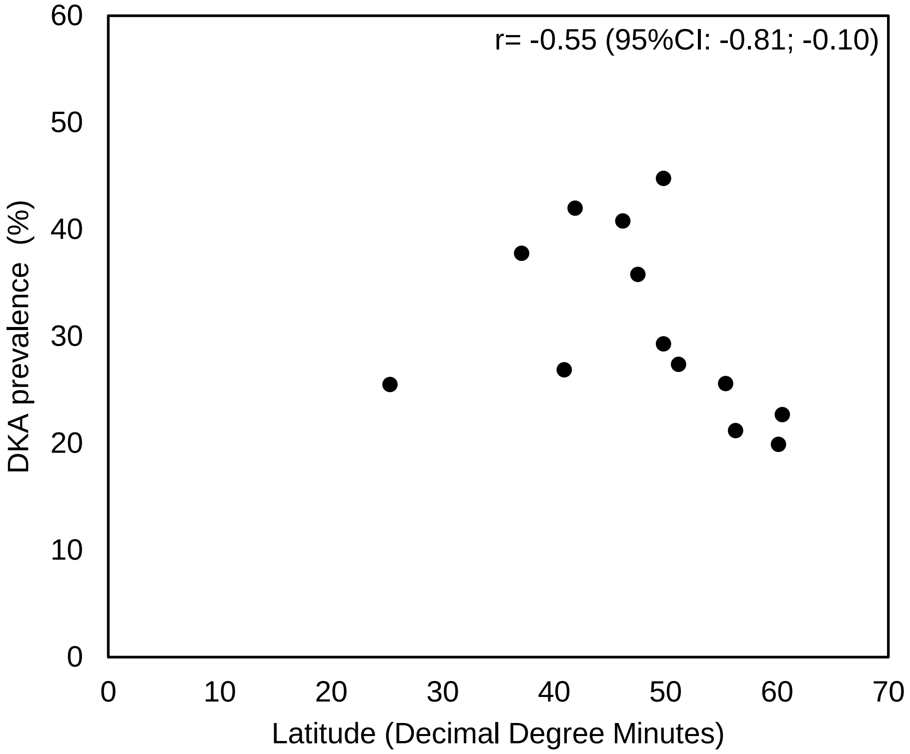

ESM Figure 5. Association between average DKA prevalence at diagnosis of type 1 diabetes, adjusted for sex and age-groups, at type 1 diabetes diagnosis (%) and Gross Domestic Product (GDP) per capita, PPP (Purchasing Power Parity, in current international thousand dollars, mean over 2006-2016), Luxembourg outlier. (**panel A**); Latitude, reported as Decimal Degree Minutes of the thirteen participating centres (**panel B**); Human Development Index (HDI, mean over 2006-2016) for the thirteen participating centres (**panel C**). Source GDP per capita, PPP and HDI from Human Development Reports by United Nations Development Programme (http://hdr.undp.org.en); source Latitude https://www.gps-coordinates.net. r refers to Spearman correlation coefficient and 95% Confidence Interval (95%CI)
